# Switching Lifestyles Is an *in vivo* Adaptive Strategy of Bacterial Pathogens

**DOI:** 10.3389/fcimb.2019.00421

**Published:** 2019-12-11

**Authors:** Stuti K. Desai, Linda J. Kenney

**Affiliations:** ^1^Mechanobiology Institute, National University of Singapore, Singapore, Singapore; ^2^Department of Biochemistry and Molecular Biology, University of Texas Medical Branch, Galveston, TX, United States

**Keywords:** acid stress, lifestyles, biofilms, SsrB, CsgD, Spo0A, chronic infections, virulence

## Abstract

Gram-positive and Gram-negative pathogens exist as planktonic cells only at limited times during their life cycle. In response to environmental signals such as temperature, pH, osmolality, and nutrient availability, pathogenic bacteria can adopt varied cellular fates, which involves the activation of virulence gene programs and/or the induction of a sessile lifestyle to form multicellular surface-attached communities. In *Salmonella*, SsrB is the response regulator which governs the lifestyle switch from an intracellular virulent state to form dormant biofilms in chronically infected hosts. Using the *Salmonella* lifestyle switch as a paradigm, we herein compare how other pathogens alter their lifestyles to enable survival, colonization and persistence in response to different environmental cues. It is evident that lifestyle switching often involves transcriptional regulators and their modification as highlighted here. Phenotypic heterogeneity resulting from stochastic cellular processes can also drive lifestyle variation among members of a population, although this subject is not considered in the present review.

## Introduction

**Figure 1:**
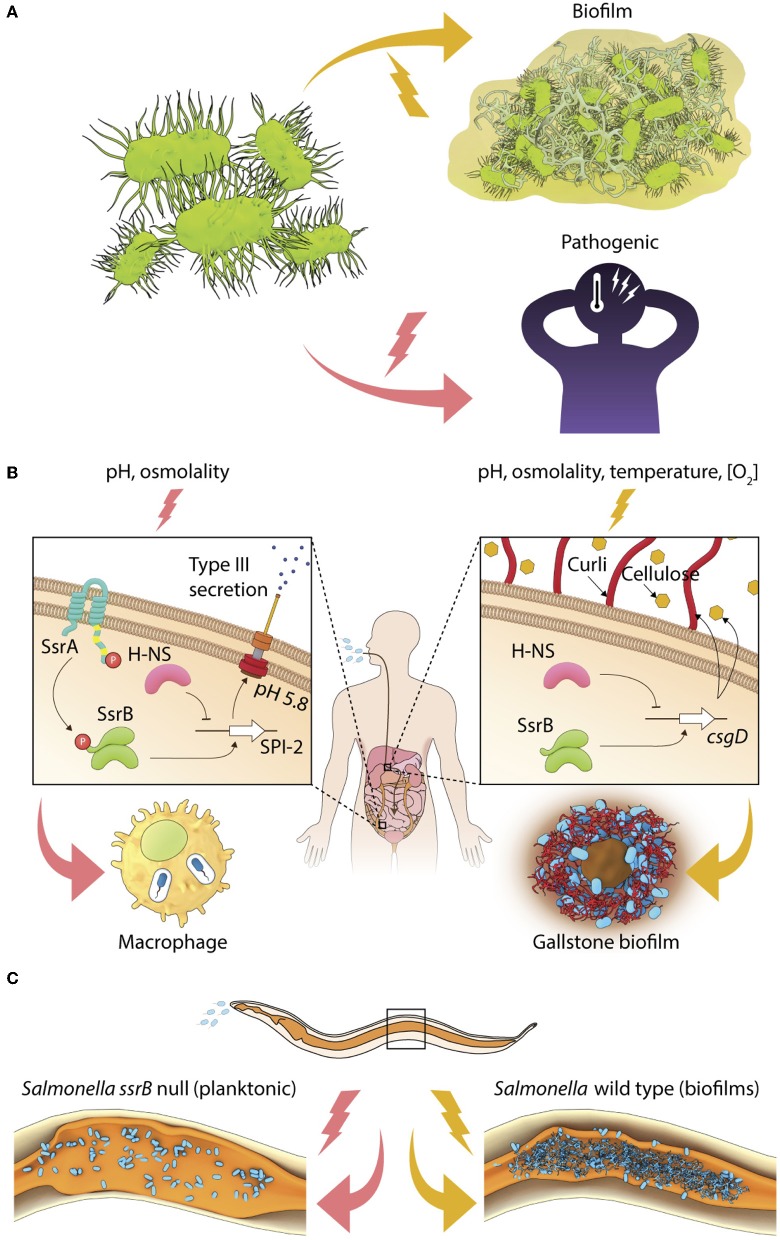
Environmental regulation of bacterial lifestyles. **(A)** A general scheme depicting lifestyle switches in pathogenic bacteria to favor virulence or biofilm formation. **(B)** In *Salmonella*, SsrB~P regulates the intracellular lifestyle and SsrB favors the formation of the carrier state and **(C)** *Salmonella* forms SsrB-dependent multicellular aggregates during persistent infections in *C. elegans*.

Pathogenic bacteria constantly face a multitude of chemical and physical stresses associated with external environments and host-specific niches. In order to survive and grow as parasites, they have evolved molecular mechanisms for altering their lifestyles in response to changes in environmental conditions. For example, free-living bacteria can switch their lifestyle to a virulent form inside hosts or undergo development to form matrix-encased aggregates called biofilms on different abiotic and biotic surfaces (Figure 1A). The virulent form rapidly colonizes and disseminates in host tissues to cause acute infections for a limited period of time. However, a prolonged association of pathogens with hosts enables carriage or persistence leading to chronic infections, which may be associated with clinical symptoms (for example, when *Pseudomonas aeruginosa* persists in the lungs of cystic fibrosis patients) or not (for example, when *Salmonella* Typhi forms biofilms on gallstones of asymptomatic carriers). We define the ability to shift from a planktonic lifestyle to a multicellular community as a “lifestyle switch,” as observed in a majority of chronic bacterial infections (Bjarnsholt, 2013). The regulation of lifestyle switches in bacterial pathogens is important to enable successful pathogenesis.

Phenotypic variations that occur in a subset of a population in the absence of any genetic or environmental drivers are classified as phenotypic heterogeneity. When this occurs, subpopulations in a clonal group adopt distinct life forms, such as planktonic or sessile, or express different functional markers, for example toxins or cell surface proteins. In this review, we address lifestyle transitions in response to changes in environmental conditions as a population trait, and do not focus on sub-populations that arise due to phenotypic or genetic heterogeneity. The exception is in the case of *Acinetobacter baumanii*, where we describe how a molecular switch gives rise to phenotypic sub-types. The adaptive significance of phenotypic heterogeneity has been superbly described in a recent review (Ackermann, 2015). In the present review, we use *Salmonella* as an example to compare with other bacterial pathogens that are known to undergo lifestyle switches.

## An Overview of *Salmonella* Pathogenesis

The enteric pathogen *Salmonella enterica* is typically ingested from contaminated food or water. Most bacteria are killed in the extreme acid pH of the stomach, but those bacteria that survive have to traverse the intestinal mucosal layer before transiting the intestinal epithelium to eventually survive inside macrophages. *Salmonella* exploits host-associated environmental cues such as acidic pH and high osmolality to form a *Salmonella*-containing vacuole (SCV), enabling intracellular replication (Lee et al., 2000; Feng et al., 2003, 2004, see Kenney, 2018, for a review). The virulence genes of *Salmonella* are encoded on horizontally acquired AT-rich segments of the genome called *Salmonella* Pathogenicity Islands (SPIs), which are tightly regulated by two-component regulatory systems (TCRSs). For example, the SsrA/B TCRS is essential for activation of the SPI-2 regulon genes encoding a type-three secretory needle and effectors that are involved in the maintenance of the SCV (Shea et al., 1996; Cirillo et al., 1998; Lee et al., 2000). Intracellular replication of *Salmonella* ultimately causes gastroenteritis (serovar Typhimurium) or systemic typhoid fever (human-restricted serovar Typhi).

Transcriptional activation of the SsrA/B system is tightly regulated by the action of upstream TCRSs, EnvZ/OmpR, and PhoP/Q, which respond to environmental changes in pH, osmolality, phosphate, and Mg^2+^ (Groisman et al., 1989; Feng et al., 2003, 2004; Liew et al., 2019). When planktonic or invasive *Salmonellae* encounter acidic pH or high osmolality, their cytoplasm acidifies, activating the membrane-bound sensor kinase EnvZ, by increasing intra-helical hydrogen bonding in its cytoplasmic domain (Wang et al., 2012; Chakraborty et al., 2015). Downstream activation of the response regulator OmpR orchestrates transcriptional activation of the SPI-2 genes, and other stress-protective mechanisms involving RpoS, the stationary-phase sigma factor, oxidoreductases, outer membrane porins, etc. (reviewed in Chakraborty et al., 2017; Kenney, 2018). SPI-2 genes are expressed when SsrB~P mediates direct transcriptional activation at SPI-2 promoters, and SsrB also functions to relieve H-NS repression (Walthers et al., 2007). Thus, coordinated activation of the sensor kinase SsrA, by mechanism(s) not precisely understood, leads to the phosphorylation of the response regulator SsrB, and enables the intra-vacuolar lifestyle of *Salmonella* in infected epithelial cells or macrophages (Figure 1B).

## The Lifestyle Switch in *Salmonella* Involves Non-Canonical Signaling

*Salmonella* also alters its genetic program to switch to a multicellular lifestyle, or biofilms, in the presence of several abiotic (for example, temperature, nutrient availability, osmolality, etc.) and biotic (for example, bile and gallbladder inflammation) stresses (reviewed in Steenackers et al., 2012). On host tissues such as gallstones and intestinal epithelial cells, individual *Salmonella* cells become encased in an intricate network of three-dimensional extracellular matrix to form mature biofilms (Boddicker et al., 2002; Crawford et al., 2010). This ability to switch to a sessile lifestyle is essential for maintaining the carrier state, allowing *Salmonella* to persist in asymptomatic patients, as well as in non-host reservoirs (Crawford et al., 2010). Studies of Typhoid carriage using the mouse model in which mice were fed a lithogenic diet to induce the formation of gallstones, have failed to provide clear insights regarding the signal transduction pathways that regulate the formation of biofilms *in vivo* or drive the switch in lifestyle from free-living cells to surface-attached communities (see Gunn et al., 2014, for a review). The transcriptional regulator CsgD in the unphosphorylated state activates the expression of biofilm matrix genes to allow the formation of *Salmonella* biofilms *in vitro* (Römling et al., 1998; Zakikhany et al., 2010; MacKenzie et al., 2015). SsrB acts non-canonically in biofilm formation (Figure 1B), in a manner that is distinct from its classical function of regulating pathogenicity island genes (reviewed in Desai et al., 2016; Desai and Kenney, 2017).

During neutral pH conditions, unphosphorylated SsrB binds to the *csgD* regulatory region and DNA binding and bending is sufficient to relieve H-NS-mediated repression, favoring formation of *S*. Typhimurium biofilms (Desai et al., 2016). Thus SsrB, a response regulator that was acquired during the evolution of *Salmonella* as a pathogen, sits at a pivotal position in governing *Salmonella* lifestyle fate: to either exist inside the host (in the SCV) as a promoter of virulence, or to drive surface-attached multicellular biofilms, which serves to maintain the carrier state (Figure 1B).

## The Adaptive Significance of Lifestyle Switching in *Salmonella*

The SsrB-driven molecular switch also functions during persistent infections *in vivo*. During *Salmonella* infection of the heterologous host *Caenorhabditis elegans*, sessile communities of *Salmonella* were clearly visible in the intestinal lumen (Desai et al., 2019 and see Figure 1C). Although the size of *Salmonella* aggregates was smaller *in vivo* (10–20 μm^2^) than a typical *in vitro* flow cell biofilm (at least 2 mm^2^), SsrB was still required, but phosphorylation of SsrB was not. The quintessential biofilm components were present, including: the master regulator CsgD, and the extracellular matrix components, curli, cellulose, and O-antigen that enabled the formation of *Salmonella* biofilms during long-term infections. Interestingly, biofilm formation enhanced the lifespan of worms, indicating a reciprocal relationship between virulence activation and the existence of biofilms. The lifestyle switch to form biofilms *in vivo* inhibited pathogenesis genes encoded on the SPI-1 pathogenicity island, and activated a mitogen-activated protein kinase (MAPK)-driven innate immunity pathway (Desai et al., 2019 and see Figure 1C). In the future, it will be important to understand the host-associated environmental cues and signal transduction pathways that activate the formation of *Salmonella* biofilms. Although we have a detailed understanding of how the SsrA/B TCRS responds to acidic pH (Liew et al., 2019), it will be germane to understand the regulation of SsrA/B expression and activity in biofilm favoring conditions.

## Lifestyle Switching in Spore-Forming Bacteria*- B. subtilis*

**Figure 2:**
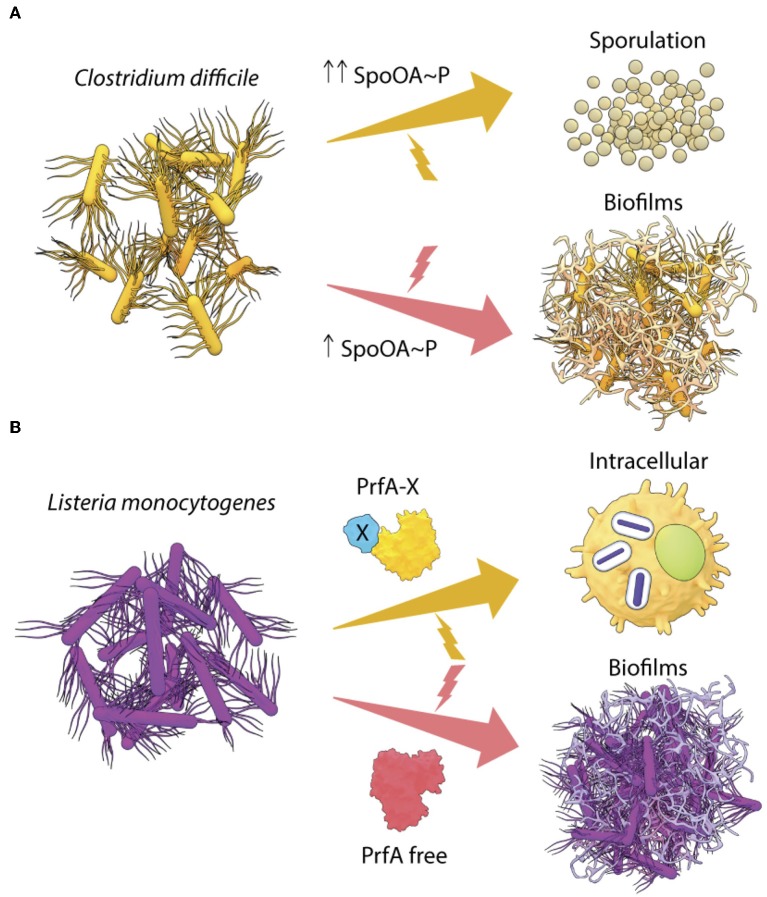
Transcriptional regulators drive lifestyle changes in Gram-positive pathogens. **(A)** In *C. difficile*, the intracellular levels of Spo0A~P regulate the lifestyle switch to form spores or biofilms and **(B)** two different forms of the transcriptional regulator PrfA in *L. monocytogenes*, are required to activate the intracellular lifestyle or to form *in vivo* aggregates.

The non-pathogenic Gram-positive bacterium *Bacillus subtilis* is an important model to understand environment driven lifestyle changes in pathogenic Gram-positive bacteria. *B. subtlis* also forms biofilms in the intestines of worms and biofilm formation increases lifespan by ~25% (Donato et al., 2017). Lifespan extension occurs when nitric oxide (NO) and Competence Sporulation stimulating Factor (CSF) produced by *B. subtilis* biofilms programs Insulin-like signaling (ILS) and MAPK innate immunity pathways of *C. elegan*s. In response to starvation, the master response regulator Spo0A orchestrates elaborate genetic changes in development and differentiation pathways in *B. subtilis* (Hamon and Lazazzera, 2001, reviewed in Vlamakis et al., 2013). In this scenario, the intracellular level of Spo0A~P controls the lifestyle decision in *Bacillus*. Intermediate levels of Spo0A~P favor biofilm formation, while a higher accumulation of Spo0A~P leads to sporulation (Fujita et al., 2005). The mature *B. subtilis* biofilm is a fine example of how heterogeneity in Spo0A~P levels leads to a division of labor, as only the matrix-producing cells differentiate to form spores (see Vlamakis et al., 2013, for a review). Such a Spo0A~P driven lifestyle switch could also be governing cell fates in the closely related anaerobe, *Clostridia*, which causes notorious nosocomial infections (Figure 2A) (see below). This is in contrast to what we observed with *Salmonella*, where unphosphorylated SsrB drove the biofilm pathway and SsrB~P was responsible for activation of virulence (Desai et al., 2016).

## Spo0A Regulates Biofilms and Sporulation in *Clostridia*

Chronic infections by the Gram-positive pathogens *C. difficile* and *C. perfringens* are highly antibiotic-tolerant and transmissible due to their remarkable ability to form hardy spores. Germination to vegetative cells leads to the production of toxins, TcdA and TcdB, adhesin, fibronectin-binding protein A, and different cell wall proteins (CWPs), which drives host colonization and disease (Waligora et al., 2001; Calabi et al., 2002; Kuehne et al., 2010; Barketi-Klai et al., 2011). In response to possible changes in temperature and nutrient levels, these free-living vegetative cells also come together and form matrix-encased biofilms (Dapa and Unnikrishnan, 2013; Obana et al., 2014). Apart from contributing to environmental persistence, biofilms of *C. difficile* have been observed on intestinal mucosal membranes of patients suffering from irritable bowel syndrome and as components of mixed species biofilms in the intestines of infected mice (Swidsinski et al., 2005; Semenyuk et al., 2015). This observation raises the question then, as to what regulates the transitions from the vegetative stage to multicellular communities and ultimately to sporulation?

Interestingly, Spo0A, the master regulator of *B. subtilis* lifestyles, is conserved in *C. difficile* with a 56% sequence identity (Deakin et al., 2012). Inactivation of Spo0A reduces biofilm formation and sporulation in *C. difficile in vitro* (Dawson et al., 2012; Dapa et al., 2013). However, complementation by Spo0A *in trans* leads to a complete recovery of the ability to form biofilms, but only to a partial rescue of sporulation (Dawson et al., 2012). Drawing parallels with what is known in *B. subtilis*, it is possible that in *C. difficile*, the activation of sporulation genes requires much higher levels of Spo0A~P than that required for biofilm formation (Figure 2A). In order to gain a clear understanding of the lifestyle switch in *C. difficile*, it would be worthwhile to examine the effect of a point mutation in the Asp66 residue (Spo0A phosphorylation site), as well as to identify the upstream kinases of Spo0A (possible homologs of *B. subtilis* KinA/B/C/D). These studies would provide insights regarding the differential regulation of Spo0A in response to environmental stresses associated with biofilms and sporulation in *C. difficile*.

## Dual States of PrfA Regulates Lifestyles of *Listeria monocytogenes*

*Listeria monocytogenes* is another Gram-positive gastrointestinal pathogen that invades and survives intracellularly in epithelial cells and macrophages to cause dangerous listeriosis in humans. The virulence program is well-characterized and requires the master transcriptional regulator PrfA. PrfA belongs to the cyclic adenosine monophosphate (cAMP) receptor protein (CRP)/fumarate nitrate reductase (FNR) family. The CRP/FNR superfamily of transcriptional regulators have evolved in several lineages of *Firmicutes, Actinobacteria, Proteobacteria*, and Cyanobacteria, and perform essential physiological functions such as catabolite repression, oxygen sensing, nitrogen fixation, and survival in stationary phase (refer to Körner et al., 2003, for a review). PrfA activates expression of the invasion factors, InlA and InlB (Gaillard et al., 1991; Dramsi et al., 1995), and the pore-forming toxin listeriolysin (LLO), enabling vacuolar escape and cytosolic replication of *L. monocytogenes* in host cells (Goebel et al., 1988; Cossart et al., 1989). The intracellular levels of PrfA are tightly controlled by feedback loops, involving post-transcriptional regulation and stress-responsive alternative sigma factors in order to ensure optimum expression during the switch from the free-living/saprophytic phase to the intracellular virulent lifestyle (see de las Heras et al., 2011, for a review). Active PrfA forms a homodimer with the C-terminus harboring a DNA-binding helix-turn-helix motif (HTH) and its N-terminus forms a β-barrel structure that was predicted to bind cyclic nucleotide(s), based on its high level of homology to the N-termini of other members of the CRP/FNR family of transcriptional regulators. However, PrfA lacks the critical cAMP-binding residues, emphasizing that sequence homology does not always predict conservation of key residues (Eiting et al., 2005). *In vivo*, transcriptional activation by PrfA is enabled by glutathione (GSH) binding (Reniere et al., 2015). Although GSH is not essential for PrfA binding to DNA *in vitro, in vivo*, the binding of GSH to each PrfA monomer stabilizes its HTH motif, and increases the probability of binding to promoters (Hall et al., 2016). Allosteric regulation of PrfA activity is also indicated by PrfA^*^ mutants that are “locked” in an active state, leading to hyper-virulence *in vivo* and a constitutive over-expression of the PrfA regulon outside the host (Ripio et al., 1996; Wong and Freitag, 2004).

Surprisingly, PrfA expression was also crucial for *L. monocytogenes* to switch to a sessile lifestyle as aggregates or biofilms (Lemon et al., 2010). The PrfA-driven pathway for formation of biofilms *in vitro* has not been worked out in detail, but studies showed that PrfA regulated biofilm maturation and growth. Interestingly, biofilms formed by the hyper-virulent PrfA^*^ mutants were similar to the wild type, however, an avirulent PrfA mutant (Y154C) formed greater biofilms *in vitro* than the wild type (Lemon et al., 2010), indicating that PrfA can exist in multiple forms with differing activities. A simple model of PrfA-mediated lifestyle switching in *L. monocytogenes* is described in Figure 2B, involving a GSH-bound form of PrfA (PrfA-X) that activates the virulence program and a free form of PrfA that drives biofilms. However, it is also possible that biofilm genes are regulated by an intermediate conformation of PrfA or an activated state bound to a different allosteric effector.

In addition, a key PrfA-regulated virulence factor ActA, also enabled *in vivo* aggregation of *L. monocytogenes* by cell-cell mediated contact in the murine infection model (Travier et al., 2013). ActA has three distinct domains, the N-terminus and P-domains are required for actin polymerization, while ActA homodimer interactions that mediate aggregate formation require N-, P-, and C-terminal domains. Thus, different active conformations of ActA might exist *in vivo* and play a role in deciding the fate of *L. monocytogenes*. An ActA-mediated lifestyle switch may also be driven by its binding to the peptidoglycan layer during the intracellular phase, although the signal(s) that enable such an association with the cell wall remain unknown (García-del Portillo et al., 2011). Interestingly, a decrease in ActA levels was also found to be correlated with the persistence of *L. monocytogenes* in vacuoles of non-phagocytic host cells (Kortebi et al., 2017). In the future, a combination of biochemical, genetic and cell biological approaches will be required to clearly delineate the structure-function relationship and regulation of expression of PrfA and ActA for favoring intracellular survival (acute phase) or persistence in hosts.

## A Novel TetR Family Regulator Switches Lifestyles in *Acinetobacter baumannii*

Phenotypic heterogeneity is the basis for several bacterial functions in specific sub-populations, including: the expression of virulence factors, quorum sensing, antibiotic resistance, and persister formation. Recent studies revealed that clinical strains of *A. baumannii* are characterized by sub-populations that differ in their cell surface properties and virulence gene expression (Chin et al., 2018). This is unlike *Listeria* (see above), in which the entire population adopts a similar morphology to become virulent or avirulent in response to niche-specific signals. *A. baumannii* cells from avirulent transparent colonies (AV-T) failed to colonize and cause disease in mice, while infections with the virulent opaque cells (VIR-O) resulted in 100% death within 2 days post infection. Transcriptomic analysis revealed that a gene encoding a TetR-type transcriptional regulator (ABUW_1645) was highly expressed in AV-T cells compared to VIR-O cells. Over-expression of ABUW_1645 in VIR-O cells reversed the phenotypic switch, leading to a loss of virulence *in vivo* (Chin et al., 2018). Since AV-T cells retained the ability to form biofilms at 25°C (a non-host temperature), biofilms and virulence might be mutually exclusive in *A. baumannnii*. How ABUW_1645 expression is regulated or whether its behavior is modified by small molecule effectors is presently not known.

## Hybrid Sensor Kinases Control the Fate of *Pseudomonas aeruginosa*

In the opportunistic Gram-negative pathogen *Pseudomonas aeruginosa*, signaling pathways that regulate virulence and biofilm lifestyles have been extensively studied. RetS is a hybrid sensor kinase/response regulator that regulates the switch between virulence and biofilms (Goodman et al., 2004). Activation of *retS* during acute infections (in response to as yet uncharacterized environmental signals), leads to the inhibition of downstream biofilm-favoring GacS/GacA/*rsmZ* signaling pathways. Since a typical DNA-binding domain has not been identified in RetS, it is not clear how RetS mediates the activation of virulence genes. Recent studies suggest that calcium may play a discriminating role. Calcium activates the periplasmic domain of LadS, a hybrid sensor kinase harboring both histidine kinase and response regulator domains, and LadS~P relays through GacS/GacA to activate the biofilm pathway (Broder et al., 2016). A similar periplasmic domain is also present in RetS, raising the possibility that calcium might inhibit the kinase activity of RetS while selectively stimulating LadS~P formation.

An interesting aspect of biofilm formation in *P. aeruginosa* is the involvement of chemosensory-type signaling by the Wsp system. Wsp signaling involves a membrane-bound methyl-accepting protein (WspA), a methyltransferase (WspC), and a methyl-esterase (WspF) which regulate phosphorylation of the response regulator, WspR, to catalyze the synthesis of cyclic-di-guanosine monophosphate (c-di-GMP) (Hickman et al., 2005). However, it is not known whether there is any cross-regulation of the Wsp system with the homologous Che proteins. It is possible that chemical stimuli might activate the sessile lifestyle in bacteria through the Che signaling system, as has been observed in the regulation of *Comamonas testosteroni* biofilms by the FlmD-CheA axis (Huang et al., 2019).

## What Regulates the Switch in Lifestyles in *Vibrio cholera*?

The ability of *V. cholerae* to enter into a non-culturable state is a major factor for environmental persistence and forms the basis of periodic cholera epidemics in endemic regions. Interestingly, aggregates of these non-culturable coccoid cells have been isolated from aquatic environments and stool samples of infected patients as matrix-encased biofilms (Alam et al., 2007). Using the rabbit ileal loop model, *Vibrio* aggregates were discovered to be hyper-virulent (Faruque et al., 2006). Although the components of *V. cholerae* biofilms are well-characterized, the signaling mechanisms that trigger biofilm formation remain unknown (reviewed in Teschler et al., 2015; Silva and Benitez, 2016). Moreover, what regulates the switch to a non-culturable state and how does temperature and salinity activate the formation of dormant cells? Detailed investigations of the TCRSs and cyclic-di-guanosine monophosphate (c-di-GMP) signaling pathways regulating *Vibrio* lifestyles in the host and outside environments will be informative.

## Concluding Remarks

A critical step for the effective targeting of bacterial pathogens is to unravel the regulatory mechanisms that govern their transitions from a free-living non-pathogenic state to a virulent state to cause disease. This is especially relevant in the present day due to rising antibiotic resistance in bacteria, frequent nosocomial infections, and a lack of novel antibiotics. In the future, the signaling mechanisms that drive the development of biofilms or multicellular communities need to be determined in actual hosts, in order to devise strategies for controlling the spread of pathogenic bacteria and eradicating chronic persistence.

## Author Contributions

SD and LK wrote the review.

### Conflict of Interest

The authors declare that the research was conducted in the absence of any commercial or financial relationships that could be construed as a potential conflict of interest.
